# Postnatal neonatal outcomes of a targeted mobile phone intervention use in antenatal care amongst pregnant women in a pastoralist community in narok county, Kenya: a randomized control trial

**DOI:** 10.4314/ahs.v24i2.26

**Published:** 2024-06

**Authors:** Daniel Muvengei, Simon Karanja, Peter Wanzala

**Affiliations:** 1 Jomo Kenyatta University of Agriculture and Technology, Public Heath; Kenya Medical Research Institute, Centre for Public Health Research; 2 Jomo Kenyatta University of Agriculture and Technology, Public Health; 3 Kenya Medical Research Institute, Centre for Public Health Research

**Keywords:** Postnatal neonatal outcomes, targeted mobile phone intervention, use in antenatal care amongst pregnant women, in a pastoralist community in Narok county, Kenya

## Abstract

**Background:**

Complications in pregnancy, at childbirth and the pueperium cause high mortality and morbidity among women and neonates globally especially in the Lower and Middle Income Countries. Antenatal care is a key high impact strategy to improve maternal and child health. The objective of the study was to examine the effects of a targeted mobile phone intervention use in the provision of antenatal care on attendance and subsequent postnatal outcomes among pregnant women in a pastoralist community.

**Methods:**

We conducted a Randomized Controlled Trial (RCT) in four hospitals in Narok County, Kenya. Pregnant women were recruited early in pregnancy and followed upto 42 days after delivery. Recruitment started in June 2018. There were two study groups; the intervention and non-intervention groups with the non-intervention group receiving the routine care.

**Results:**

Two-hundred-and-sixty-two of the 280 study participants completed the study (93.6% response rate). The difference in proportion of study participants who had neonatal mortality at birth between the two study arms was 9.32% (95% CI 1.91-16.74%) between the intervention (6.06%) and the non-intervention (15.38%) study arms (p value = 0.015).

**Conclusion:**

A targeted mobile phone intervention used in antenatal care was associated with improved antenatal care attendance and better neonatal outcomes.

## Background

Complications occurring during pregnancy, at child birth and the immediate postnatal period are the leading causes of mortality and morbidity among Women of Reproductive Age (WRA) and neonates in the world^1^. Most women and neonates die because of complications occurring during and following pregnancy, which include but not limited to hemorrhage, hypertension and infections^2^. The majority of the mortality and morbidity is due to preventable causes and almost all of it (99%) occurs in Lower and Middle Income Countries (LMICs)^3^.

Neonatal Mortality Rate (NMR) in Kenya was 22 deaths per 1000 live births in 2014 4. The rate was 27 deaths per 1000 live births in 1990 5. Globally, the rate was estimated at 19.2 deaths per 1000 live births in 2015 5. The rate of improvement has been slow in Kenya. NMR is a key and sensitive outcome indicator of the quality of healthcare being offered in a country. It directly reflects prenatal, intrapartum, and neonatal care 6. Early neonatal deaths (within first 7 days of life) are more closely associated with pregnancy-related factors and maternal health, whereas late neonatal deaths (after the 7th day of life) are associated more with factors in the newborn's environment 6.

Antenatal care (ANC) is one of the key high impact strategies to improve maternal and child health globally^1^. World Health Organization (WHO) currently recommends at least 8 visits during the woman's pregnancy^7^,^8^. In low and middle income countries, only about half of pregnant women receive the WHO recommended minimum ANC visits^9^. It is also recommended that women should have at least one or more postnatal visits within 28 days of delivery^10^,^11^. Countries that have well developed and quality antenatal and postnatal care services and where the ANC attendance by the population is high have impressive maternal and neonatal outcomes. For example in Finland where there is almost universal ANC attendance (99.8%), NMR is 1 death per 1000 live births^12^. Good quality ANC reduces maternal morbidity and mortality and perinatal morbidity^3^,^13^. Early initiation of ANC and attendance of more visits are associated with higher infant birth weights and lower infant mortality rates.^14^,^15^.

Evidence exists to show that using technology in maternal health (mHealth) improves outcomes^15^. Studies show that mobile phone technology is effective at changing behaviour to improve antenatal care and postnatal care attendance^15^. However most of the studies in the literature are observational and more rigorous evaluation of mHealth is necessary in a broader variety of settings. The mobile phone subscriptions in Kenya was 53.2 million in 2019 compared to the official 2019 population of 47.5 million Kenyans translating to mobile (SIM) penetration level of 112.0 percent 16. Leveraging on this high mobile phone penetration within the country to improve healthcare services and outcomes is critical.

### Problem Statement

Across sub-Saharan Africa, there is wide variation in ANC attendance^6^. Seventy four percent of pregnant women attend formal ANC at least once during their pregnancy^6^. However, only 44% of women attend ANC four or more times^4^,^5^,^6^. The Kenya Health Demographic survey (KDHS) 2014 reported that 9 in 10 mothers saw a skilled provider at least once for ANC for their most recent birth in the five-year period before the survey^4^,^15^. However, only 58% of women reported 4 or more visits^15^. This proportion varies widely across counties with West Pokot County reporting 18.2% while Nairobi County reported 73%^15^. Narok County reported 46.0% fourth ANC attendance^15^.

The KDHS 2014 reported that in Narok County only 40% of the deliveries were assisted by a skilled healthcare attendant and only 39% of these deliveries were in a health facility, which was much lower than the national achievements of 62% and 61% respectively^4^. The low proportion of attendance of ANC has been correlated with poor prenatal and postnatal outcomes in Africa with maternal and infant morbidities and mortalities remaining high^17^,^18^. Those mothers who attend more ANC visits are also more likely to deliver under a skilled healthcare attendant^17^.

Socio-cultural challenges of ANC coupled with inadequate services being offered in health facilities make the impact of antenatal services much less than would be expected^18^. Lower literacy levels in some parts of Kenya make maternal and child healthcare delivery more complicated and the health outcomes less than favourable^15^.

The field of mHealth and more specifically mobile technology is proposed as a potential solution to the many challenges facing middle and low income countries in health care delivery^19^. Text messages have been shown to improve health seeking behaviour, treatment adherence, data collection and as a communication tool to improve patient follow up and data reporting^20^,^21^,^22^. Given that mHealth tools have been promising in behaviour change broadly, potential exists to improve essential preventive maternal and child health services as well.

Most studies that have been done to examine causes and solutions of low ANC attendance have largely been observational and few interventional studies have been done especially in Kenya to examine low-cost and widely scalable innovative mobile interventions to improve ANC attendance^4^,^5^,^6^,^23^. These studies have utilized Short Message Services (SMS) with only one study adding a voucher component to the SMS^23^. Many existing interventions also focus on single component of maternal and child health preventive services instead of a design of integrated system that follows women and children through the maternal, neonatal and child health continuum^24^.

This study adds to the armament of available interventions a bi-component intervention that can be used to improve antenatal care attendance and help reduce the morbidity and mortality associated with childbearing in Narok County, Kenya. The findings of this study will be useful to the Narok County's Department of Health in planning for maternal and child health interventions. The community will also benefit by reduction in the mortality and morbidity associated with pregnancy. The national government of Kenya's Ministry of Health can use the findings of this study in policy formulation on maternal and child health too.

### Study objective

The main objective of the study was to examine the effects of a targeted mobile phone intervention use in the provision of antenatal care on attendance and the subsequent maternal and neonatal postnatal outcomes among pregnant women in a pastoralist community in Narok County. We hypothesized that use of a targeted mobile phone intervention in antenatal care would not be associated with improved maternal and neonatal outcomes and few complications.

## Methods

### Study Site

This study was conducted in four hospitals in Narok County; the Narok County Referral Hospital, a level 5 county referral hospital, Ololunga Sub-county Hospital, a level four sub-county hospital, Ntulele and Mulot Health Centres, both busy level three hospitals. Narok County is one of the 47 sub-national governments in Kenya located on the southern part of Kenya bordering Kajiado county to the east, Nakuru county to the north, Bomet, Nyamira, Kisii and Migori counties to the West and the People's Republic of Tanzania to the South. It had an estimated population of 1.28 million in 2022. The average household size was five members^25^. The Total Fertility Rate was estimated at 4.7 per woman in 2019^25^. The study population comprised the women of reproductive age (WRA) who were expectant and were attending antenatal care at the Maternal and Child Health Clinics in the study facilities.

### Study Design

A Randomized Controlled Trial (RCT) was conducted to determine the effects of the targeted mobile phone intervention on antenatal care attendance, level of skilled attendant delivery and the resultant postnatal maternal and neonatal outcomes. Pregnant mothers were recruited early in pregnancy within their first or second trimester, enrolled on providing informed consent and followed up for up to 42 days after delivery. Recruitment started in June 2018. The targeted mobile phone intervention was administered on the women in the intervention study arm while those in the non-intervention arm received the routine/usual antenatal care.

The intervention had two components; firstly was a standardized Short-message Service (SMS) designed to include health education on importance of antenatal care attendance and a reminder to attend the ANC clinics regularly that was sent fortnightly using an individualized messaging system immediately after recruitment. The second component was a phone call reminder that was done one week before the date the study participant had been booked to attend the ANC clinic. Bookings were done on a monthly basis from the date of recruitment.

Two research assistants were recruited at each participating facility, trained on the study protocol and tasked with implementation of the study which involved recruitment, enrolment, timely sending of the standard SMS and timely calling of these study participants. This information was maintained in study registers. They were also involved in the clinical follow up of the mothers and patient record management in the clinics during the study period.

After recruitment the research assistants would immediately inform the principal investigator (PI) about the recruited study participant. The PI would then allocate the newly recruited mother to their specific study arm using a generated list of random numbers and then relay this information back to the assistants who would then record this in the study registers and depending on the study arm to which the mother fitted in, commence the intervention immediately.

The primary postnatal neonatal outcomes of interest were measured at birth in the maternity wards and were extracted and recorded in the study participant record form upon checking in the facility postnatal registers. These primary neonatal outcomes included status of baby at birth, APGAR score, birth weight, any complications at birth including neonatal mortality. Those study participants who were not found in the facility registers, their data were extracted from the MOH-approved antenatal booklets when the mothers visited the postnatal clinics for immunizations. Those study mothers who did not deliver in any of the participating health facilities and did not come for immunizations were called using the phone numbers they had provided at recruitment and the outcomes of interest recorded in the study participant record form.

### Selection criteria

Recruitment of study participants was done on the basis of an expectant mother, the mother attending her first ANC within the first or second trimester and also owning a mobile phone. In cases where the mother did not have a mobile phone, a contact within the household was required to own a phone e.g. spouse or parent. Study participants were also required to have been resident in the county of Narok for at least 5 years prior to recruitment. Minors were required to have a caregiver who would give informed consent. Participants who did not give consent and those who had co-morbidities were excluded from the study.

### Sample size calculation

The alpha and the power of the study were assumed to be 0.05 and 80% respectively. The study arms were of equal size. The proportion of deliveries assisted by a skilled healthcare attendant in Narok was estimated at 0.40 by the KDHS 2014^4^. This study assumed a clinically significant improvement of 0.20 making this proportion 0.60 (national average). Using the Fleiss' formula, the calculation gave a sample size of 107 ANC mothers in each study arm. A 10% and 18% adjustment were done for dropouts and contamination respectively giving a total sample size of 140 per study arm^26^.

### Data collection

Data were collected using a study participant record form, which had been divided into different sections with each section being filled at different times in the course of the pregnancy and the study. Section A contained socio-demographic data; section B, anthropometric and systemic clinical examination. Section C contained had postnatal outcomes information extracted from registers and section D contained an qualitative assessment of the acceptability of the intervention.

### Data Analysis

Data Analysis was done using Stata statistical Software v14. Quantitative data were collected. Data were presented in form of tables, charts and graphs. Descriptive statistics using means, medians and standard deviations were used. Associations and hypotheses were tested using independent t tests and Chi Square. For non-normally distributed variables, non-parametric tests of significance (Mann Whitney U) tests were used. Further statistical analysis to develop a model was done using logistical regression analysis to determine the predictors of neonatal mortality in this population.

### Ethical approval

Ethical approval was obtained from the Kenya Medical Research Institute's Scientific and Ethics Review Unit (SERU) protocol number KEMRI/SERU/CPHR/001/3573.

## Results and discussion

### The Organization of the Randomized Control Trial (RCT)

**Figure 1:**
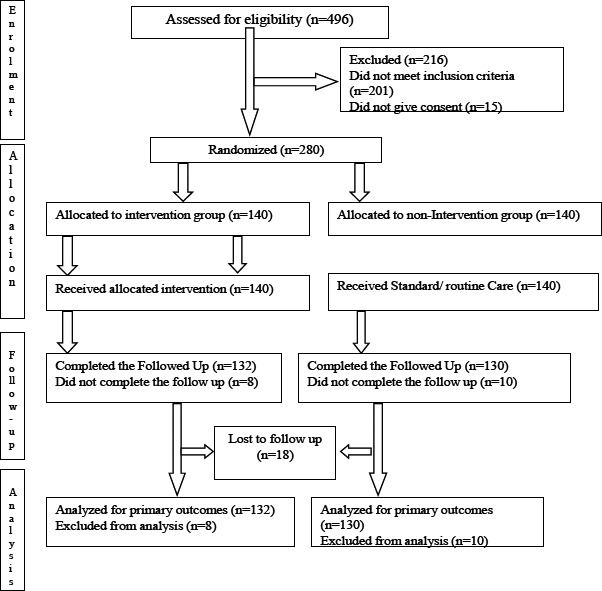
Flowchart of Phases of Parallel Randomized Trial–Modified from CONSORT 2010

The flowchart (Figure 1), derived from the CONSORT 2010^27^ guidelines on clinical trials, shows the study flow. Two hundred and sixty two out of the two hundred and eighty (280) study participants completed the study, giving a response rate of 93.6%. The baseline characteristics are shown in table 1. These characteristics showed that the study participants in the two study groups (intervention and the non-intervention) were comparable by all the characteristics at baseline. Majority of them were married (86%) and had attained up to secondary school level of education.

**Table 1:** Table showing the baseline characteristics of study participants

Variable	Intervention Arm (N=132)	Non-Intervention Arm (N=130)
Age (Y ears)	24.29 ± 5.29	23.44 ± 5.12
Marital Status: n (%)		
Married	115 (87.12)	111 (85.38)
Single	16 (12.12)	19 (14.62)
Separated	1 (0.38)	
Parity (Number of children)	1 ± 1.03	0.88 ± 1.29
		
Level of Education: n (%)		
Never Attended		7 (5.38)
Primary	47 (35.61)	47 (36.15)
Secondary	53 (40.15)	47 (36.15)
Tertiary	32 (24.24)	29 (22.31)
		
Level of Education attained by Spouses: n (%)		
Never Attended	1 (0.76)	4 (3.08)
Primary	30 (22.73)	30 (23.08)
Secondary	47 (35.61)	48 (36.92)
Tertiary	38 (28.79)	29 (22.31)
		
Distance to a Health Facility: n (%)		
Less than 1 km	24 (18.18)	19 (14.62)
1 to 5 km	74 (56.06)	90 (69.23)
More than 5 km	34 (25.76)	21 (16.15)
		
Time taken to a Health Facility: n (%)		
Less than 15 minutes	20 (15.15)	12 (9.23)
15 to 30 minutes	38 (28.79)	35 (26.92)
30 to 60 minutes	56 (42.42)	74 (56.92)
More than 60 minutes	18 (13.64)	6 (6.92)
		
Spouses' Drinking/Smoking status: n (%)		
Didn't Drink or Smoke	95 (83.33)	92 (84.4)
Drank and/or Smoked	19 (16.67)	17 (15.6)
		
Key Decision Maker at Family Level: n (%)		
Couple together	41 (31.06)	41 (31.54)
Husband	33 (25)	32 (24.62)
Study Participant	53 (40.15)	51 (39.23)
Parent	4 (3.03)	5 (3.85)
Mothers with Previous Scar: n (%)		
With Scar	13 (9.85)	8 (6.15)
Without Scar	119 (90.85)	122 (93.85)
		
Height (Metres)	1.578 ± 0.066	1.57 ± 0.061
Weight (Kg)	61.35 ± 10.48	60.45 ± 10.79
BMI	24.92 ± 4.52	24.62 ± 4.77
Systolic BP (mmHg)	116.90 ± 13.57	114.90 ± 12.38
Pulse Rate	78.81 ± 6.82	79.73 ± 7.75
Temperature (^0^)	36.63^0^±0.37	36.61^0^± 0.43
Gestation at Enrolment by Fundal Height (Weeks)	19.43 ± 4.94	20.09 ± 4.39
Gestation at Enrolment by Dates (Weeks)	19.53 ± 6.44	20.13 ± 4.87
Time of Follow (Weeks)	20.59 ± 6.02	19.78 ± 5.04
Hemoglobin (g/dl)	11.75 ± 1.71	11.57 ± 1.46
		

Values are Means ± SD unless otherwise indicated

a Numbers may not add up to 132 or 130 due to missing values

### Study Participants Age at Enrolment

The overall mean age was 23.87 years, with the minimum age of a study mother being 15 years and the maximum age being 44 years giving a range of 29 years. There were 119 study participants aged between 20 and 24 years accounting for the majority of the study mothers at 45.42%, followed by the 59 mothers aged between 25 and 29 years at 22.52%. The 50 study mothers aged 19 years and below (teens) were the third highest at 19.08%. The least proportion of study participants were those aged 40 years and above forming 1.13%.

### The Intervention

The mean number of SMS sent to the 132 study mothers in the intervention arm was 11.93 messages (SD 2.50, 95% CI 11.50-12.36). The median number of SMS sent was 12 messages and a range of 15 messages. The mean number of calls done to the study mothers was 4.68 calls (SD 1.24, 95% CI 4.47-4.89). The median number of calls done was 4.5 calls and a range of 6 calls.

### Number of ANC Visits by Study group

The mean number of ANC visits was 4.099 visits for the intervention group while it was 2.843 visits for the non-intervention group giving a mean difference of 1.256 visits (95% CI 1.044-1.467) which was statistically significant with p value of <0.0001 (A different publication)

### Neonatal Outcomes

The main neonatal outcomes of interest are shown in the table below (Table 2).

**Table 2:** Table showing the Neonatal Outcomes by Study Group

Variable	Intervention	Non-Intervention	P Value
**Status of baby at delivery (%, n):** ○ Cried Immediately ○ Cried after a few minutes ○ Did not cry at all ○ Did not move at all			
	86.15 (112)	64.84 (83)	
	12.31 (16)	30.47 (39)	
	0.77 (1)	3.13 (4)	
	0.77 (1)	1.56 (2)	
**APGAR Score (Mean, SD, 95% CI)**	9.36 (0.83, 9.21-9.51)	8.33 (2.20, 7.89-8.77)	<0.0001
**Birth Weight (Mean, SD, 95% CI)**	3228 grams (442, 3149-3307)	3252 grams (514, 3154-3350)	0.7036
**Neonatal Complications (%, n)**			
Yes	12.21% (16)	28.68% (37)	**<0.0001**
No	87.79% (115)	71.32% (92)	
**Neonatal Mortality (%, n)**			
Yes	6.06% (8)	15.38% (20)	**0.015**
No	93.94% (124)	84.62% (110)	
			

### APGAR Score at 5 Seconds by Study Group

The distribution of the mean APGAR score at 5 seconds was skewed to the left hence Mann Whitney U test of significance was used due to this skewness to test the null hypothesis that there was no difference between the median APGAR scores at 5 seconds of the two study groups. The difference was found to be statistically significant at 95% confidence level (z = 4.698, p value < 0.0001) thus the null hypothesis was rejected. This meant that the babies of the study participants in the intervention arm were more likely to have higher APGAR Scores at birth compared to those in the non-intervention group.

### The Birth Weight by Study Groups

**Figure 2:**
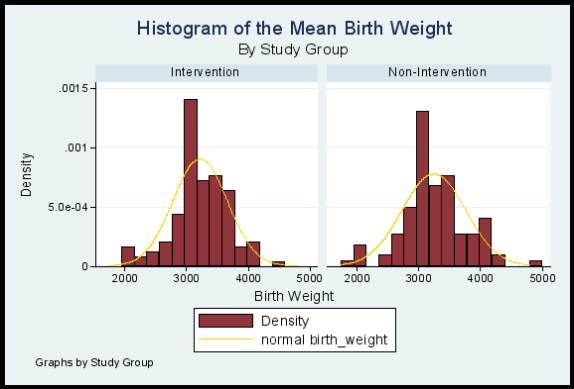
Histogram showing the Distribution of the Mean Birth Weight by Study Group

The birth weight of study participants in the two study arms was normally distributed around the two means as shown in the histogram (Figure 2). The difference in means was 24.13 grams (95% CI -148.98 to 100.71). An independent t-test of significance to test a null hypothesis of there being no difference between the mean birth weights of the two study arms found it not to be statistically significant at 95% confidence level (p value = 0.7036). Thus the null hypothesis was not rejected, meaning that the babies in the two study groups had comparable weight at birth.

### Neonatal Complications

The overall prevalence of neonatal complications was 20.38% (n=53) while 79.62% (n=207) of the babies had no complications at birth. The commonest reported neonatal complication was birth asphyxia at 60.38% (n=32), followed by low birth weight at 13.21% (n=7).

**Figure 3:**
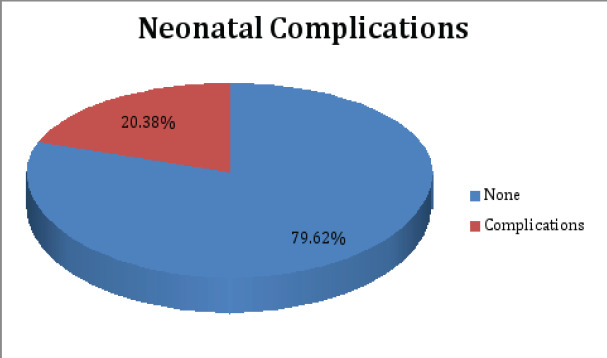
Pie Chart showing the proportion of study participants babies with Complications at Birth

Fetal distress and fresh stillbirth (FSB) constituted 5.66% (n=3) respectively. Two babies had very low birth weight (VLBW) constituting 3.77% of all reported neonatal complications while one baby each had neonatal sepsis, post-datism and prematurity constituting 1.89% respectively. The prevalence is depicted in the pie chart below (Figure 3)

### Neonatal Complications by Study Group

A t-test of proportion was used to test the difference in proportion between the two study groups of study mothers who had a neonatal complication at birth. The difference in proportion was 16.47% between the non-intervention (28.68% (n=37) and intervention group (12.21% (n=16). The difference was statistically significant with a p value of less than 0.0001. This meant that the study participants in the non-intervention group were more likely to experience a neonatal complication compared to those in the intervention study group.

### Neonatal Mortality

**Figure 4:**
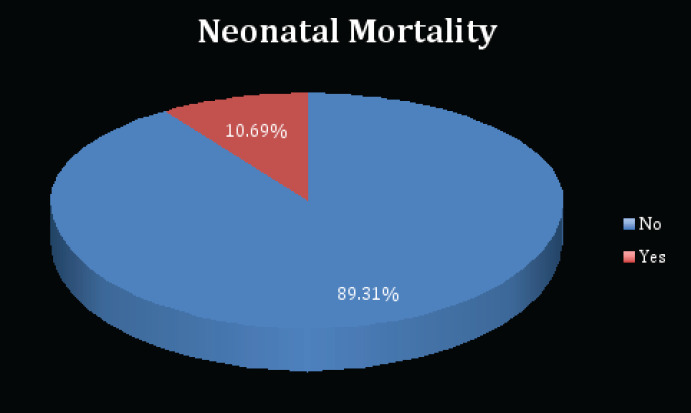
Pie Chart showing the Proportion of Study Participants who had a Neonatal Mortality

Of the 262 study participants, 89.31% (n=234) had live births while 10.69% (n=28) had neonatal deaths. This is depicted in the pie chart below (Figure 4).

### Neonatal Mortality by Study Group

A test of significance using t-test of proportions of the null hypothesis that there was no difference in the proportion of study participants who had neonatal mortality at birth between the two study arms was done. This difference was 9.32% (95% CI 1.91-16.74%) between the intervention (6.06%) and the non-intervention (15.38%) study arms and was statistically significant at 95% level of confidence (p value = 0.015). Thus the null hypothesis was rejected meaning that the mothers in the non-intervention arm were more likely to have more neonatal mortalities compared to the intervention study arm.

### Neonatal Outcomes by Place of Delivery

Neonatal outcomes were also examined by the place of delivery and are summarized in the table below (Table 3)

**Table 3:** Table showing the neonatal outcomes by place of delivery

	Hospital	Home	P value
**Status of baby at delivery (%, n):** ○ Cried Immediately ○ Cried after a few minutes ○ Did not cry at all ○ Did not move at all			
	82.52 (170)	48.08 (25)	
	14.56 (30)	48.08 (25)	
	1.46 (3)	3.85 (2)	
	1.46 (3)		
**Neonatal Mortality (%, n)**			
**Yes**	3.88% (8)	30.77% (16)	**<0.0001**
**No**	96.12% (198)	69.23% (36)	
			

### Neonatal Mortality by Place of Delivery

**Figure 5:**
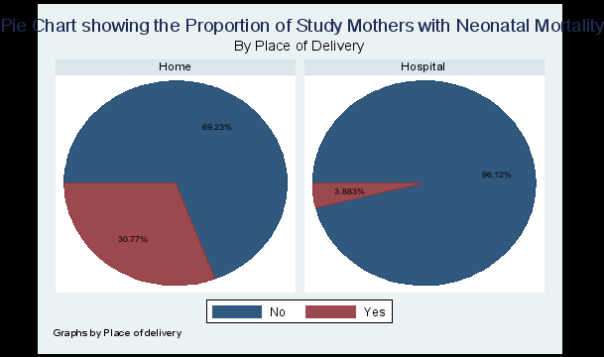
Pie chart showing the proportion of study mothers with neonatal mortality by place of delivery

For the 52 study participants who delivered at home, 69.23% (n=36) of the study mothers had no neonatal mortality while 30.77% (n=16) had a neonatal death. For the 206 study mothers who delivered in hospital, 96.12% (n=198) of the study mothers had no neonatal mortality while 3.88% (n=8) had a neonatal death. This is summarized in the pie chart (Figure 5). A t-test of the difference in the proportion of the study participants who had a neonatal mortality by place of birth showed that the difference of 26.89% between the two groups was statistically significant at 95% statistical significance with a p value of less than 0.0001 implying that the babies of the mothers who delivered at home were more likely to suffer from a neonatal mortality compared to the babies of the mothers who delivered in the hospital.

### Regression Modeling

A logistic regression model of the predictors of the likelihood of the baby of a study participant experiencing a neonatal mortality was developed. In order to build this model, the dependent variable was regressed with all the independent variables bivariately in order to check for their statistical significance. Then the statistically significant independent variables formed the basis for the subsequent multivariate regressions. The results of bivariate regression are shown in table 4.

**Table 4:** Table showing the Regression Results of the Dependent Variable and the Independent Variables (Bivariate Regression)

Dependent variable	Covariate	Odds Ratio	P-value	Confidence Interval (95%)	McFaddens' R^2^
Neonatal Mortality	None	0.1196	0.000	0.0808-0.1771	0.0000
	Age	1.0319	0.386	0.9612-1.1077	0.0041
	Parity	1.3379	0.050	1.0002-1.7898	0.0200
	BMI	1.0933	0.035	1.0062-1.1878	0.0285
	SBP	1.0002	0.895	0.9723-1.0327	0.0001
	Gestation_FH	1.0929	0.063	0.9951-1.2003	0.0219
	Hemoglobin	1.1013	0.495	0.8347-1.4531	0.0033
	Place_del	0.0901	0.000	0.0362-0.2281	0.1743
	Assist_Del-2	10.4210	0.000	3.5136-30.908	0.1745
	Assist-3	11.6471	0.000	3.8845-34.922	
	NoofANCVisits	0.2553	0.000	0.1532-0.4252	0.2340
	Mode_del	0.7254	0.582	0.2314-2.2738	0.0019
	NoofSMSsend	0.5985	0.003	0.4268-0.8393	0.1817
	NoofCallsDone	0.3210	0.007	0.1406-0.7329	0.1567
	Dates at Enrol	1.0072	0.216	0.9958-1.0187	0.0103
	DaysofFollowup	0.9895	0.228	0.9727-1.0066	0.0181
	Study Group	2.8182	0.018	1.1935-6.6542	0.0344
	Hosp Level 1	3.1590	0.056	0.9688-10.299	0.0217
	Level 2	1.9576	0.211	0.6836-5.6058	
	Maritalstatus	0.2168	0.140	0.0285-1.6486	0.0193
	Educ_level2	0.0893	0.004	0.0174-0.4576	0.0547
	level3	0.0742	0.002	0.0143-0.3848	
	level4	0.0670	0.003	0.0116-0.3870	
	Distance_Hosp	1.0297	0.952	0.3958-2.6786	0.0000
	Time_Hosp	1.7701	0.193	0.7489-4.1837	0.0101

The following variables were statistically significant on bivariate regression; BMI (OR 1.093, 95% CI 1.006-1.188, p=0.035); place of delivery (OR 0.090, 95% CI 0.036-0.228, p<0.0001); assistant at delivery (OR 10.421, 95% CI 3.514-30.908 for relative, OR 11.647, 95% CI 3.885-34.922 for traditional birth attendant, p<0.0001); study group (OR 2.818, 95% CI 1.194-6.654, p=0.018); number of ANC visits (OR 0.255, 95% CI 0.153-0.425, p<0.0001); number of SMS sent (OR 0.598, 95% CI 0.426-0.839, p=0.003); number of calls done (OR 0.321, 95% CI 0.141-0.733, p=0.007) and education level (OR 0.089 95% CI 0.017-0.458 p=0.004 for primary education, OR 0.074, 95% CI 0.014-0.385, p value = 0.002 for secondary education and OR 0.067, 95% CI 0.012-0.387, p=0.003 for tertiary education).

Multivariate regression was done with the statistically significant independent variables and clinically significant independent variables to develop a statistical model that could show the predictors of neonatal mortality in this population and after several iterations the model with statistically significant independent variables and age was found to be the best fitting with a p-value of 0.0006 and a McFadden's R2 of 0.7003. The Akaike Information Criteria (AIC) and the Bayesian Information Criteria (BIC) for this model were 24.11 and 42.49 respectively. This information is depicted in table 5. The independent variables in the chosen model were assessed to check for any interactions between them and none of the interaction terms was found to be statistically significant.

**Table 5:** Table of the Logistic Model of Neonatal Mortality with the Statistically Significant Covariates and Including Age

Dependent variable	Covariate	Odds Ratio	p-value	Confidence Interval (95%)	McFaddens' R^2^	Model p-value	AIC & BIC
Neonatal Mortality	Age	0.2477	0.132	0.0403-1.5245	0.7003	0.0006	24.11 42.49
	BMI	5.0110	0.131	0.6178-40.643			
	Place del	142361	0.187	0.0032-6.33*10^12^			
	No of ANC Visits	0.0564	0.143	0.0012-2.6334			
	No of SMS Sent	0.0507	0.137	0.0010-2.5692			
	No of Calls done	0.0433	0.215	0.0003-6.1809			
	Constant	6.93*10^10^	0.170	0.00002-2.07*10^26^			
	Hosmer-Lemeshow Chi Square statistic = 3.72 and p value = 0.8817						

### Marginal Plots

**Figure 6:**
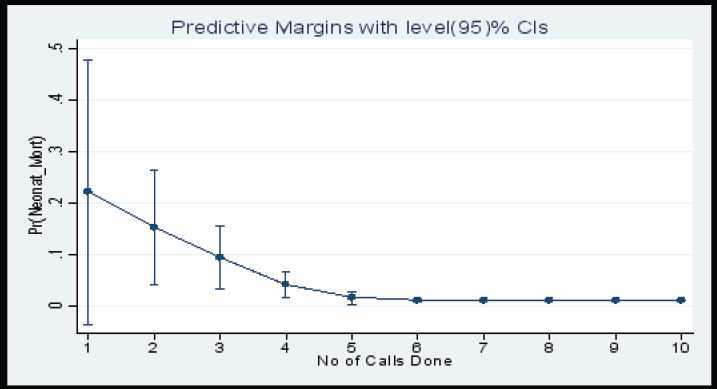
Marginal Plot of the Probability of having a Neonatal Mortality by Number of Calls Done to Study Participants

**Figure 7:**
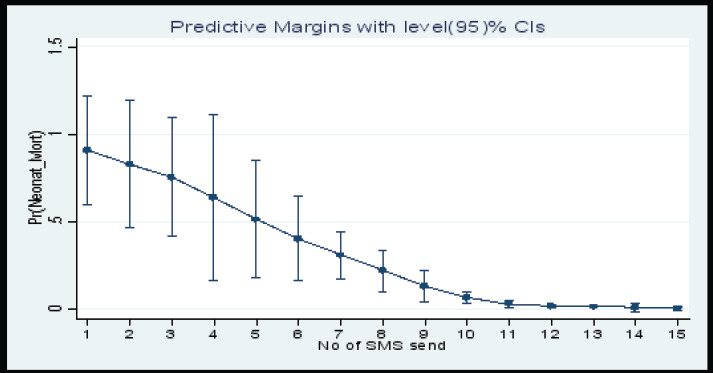
Marginal Plot of the Probability of having a Neonatal Mortality by Number of SMS Sent to Study Participants

This marginal plot (Figure 6) for the number of calls done showed that the probability of having a neonatal mortality decreased as the number of calls done to the study participant increased up to six calls and then remained constant there after. Whereas the marginal plot (Figure 7) for the number of SMS sent showed that the probability of having a neonatal mortality decreased as the number of SMS sent to the study participant increased up to twelve SMS and then remained constant there after.

### Prediction Plots

**Figure 8:**
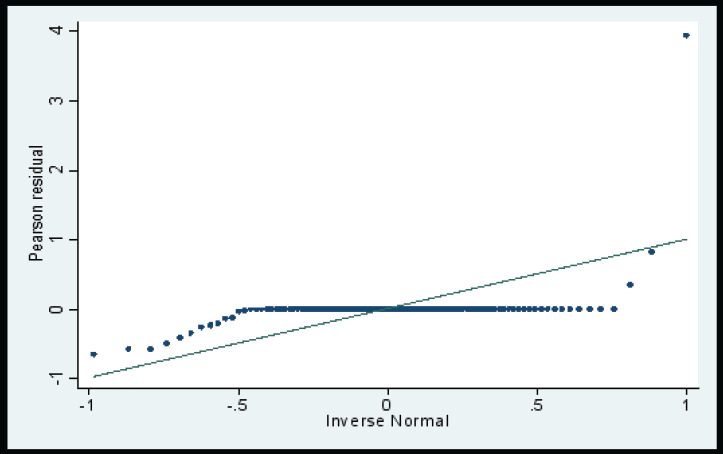
Q Norm Plot

The residual plots (Q Norm Plot) (Figure 8) showed that the developed model was a good predictor of the dependent variable.

## Discussion

Several studies have studied effects of components of mHealth applications/tools on antenatal care. However most of the studies have focused on single components (majorly SMS) and single endpoint-ANC attendance^28^-^34^. A cluster RCT study done in Zanzibar by Lund S et al was bi-component studying SMS plus a voucher system to improve ANC attendance^34^. The study showed that the SMS and voucher system was effective in improving ANC attendance by 44%^34^. Having been a cluster RCT the challenges of selection and dilution bias was possible. This study did not also follow up the study participants to examine the postnatal outcomes of the intervention among the study population for comparison with the non-intervention study group.

In the current study, the prevalence of neonatal complications was lower in the intervention group (12.21%) compared to the non-intervention (28.68%). The difference in the proportion was statistically significant with a p value of less than 0.0001. The babies in the intervention group also had comparatively better APGAR scores (Mean of 9.36) compared to the non-intervention group (mean of 8.33) with a statistically significant difference. The study participants in the intervention study group were also less likely to have neonatal mortality compared to those in the non-intervention group. The difference in proportion of the likelihood of a study participant having a neonatal mortality at birth was 9.32% (95% CI 1.91-16.74%) between the intervention (6.06%) and the non-intervention (15.38%) study arms, which was statistically significant at 95% level of confidence (p value = 0.015).

On bivariate logistic regression, the number of calls done (OR 0.321 95% CI 0.1406-0.7329, p value = 0.007) and the number of SMS send (OR 0.5985 95% CI 0.4268-0.8393, p value = 0.003) were statistically significant predictors of likelihood of having a neonatal mortality and were both protective, meaning that the more SMS's and calls done to participants the lower the likelihood of having a neonatal mortality. Combining these two components to improve ANC attendance and thus subsequent postnatal outcomes was useful. The assistant at delivery was also a statistically significant predictor of likelihood of having a neonatal mortality with the odds of neonatal mortality multiplied by a factor of 10.421 (95% CI 3.5136-30.908) for those assisted by a relative and 11.6471 (95% CI 3.8845-34.922 for those assisted by a traditional birth attendant compared to a healthcare worker. These were high odds indicating the need for deliveries to be conducted by skilled healthcare workers.

The developed multivariate logistic regression model for predictors of neonatal mortality showed that the model was statistically significant (p value = 0.0006), had good McFadden's R2 of 70.03% and AIC and BIC of 24.11 and 42.49 respectively and was a good fit for the variables (Hosmer-Lemeshow Chi Square statistic). All these indicated a robust model for determining predictors of neonatal mortality in this population.

## Conclusion and recommendations

A targeted mobile phone intervention used in antenatal care was associated with improved antenatal and postnatal care attendance and better neonatal outcomes with fewer complications.

## Limitations of the study

The main limitation of the study was that the study participants (first ANC visit) were recruited from the MCH clinics and thus these mothers could possibly be different from the general population since they had already come to start their ANC visits early since most of our mothers attend ANC late. A community based recruitment model would potentially minimize this bias with attendant higher study costs.
